# CREATING AND SUSTAINING EXTENSION PARTNERSHIPS TO REACH RURAL OLDER ADULTS: THE STAY STRONG STAY HEALTHY PROGRAM

**DOI:** 10.1093/geroni/igae098.1701

**Published:** 2024-12-31

**Authors:** Kristin Miller, Kelsey Weitzel, Naomi Meinertz

**Affiliations:** University of Missouri, Columbia, Missouri, United States; University of Missouri, Columbia, Missouri, United States; University of Missouri, Columbia, Missouri, United States

## Abstract

Due to out-migration of family members and a lack of transportation and access to resources, rural-dwelling older adults often experience greater social isolation, which is linked to worse health outcomes and a loss of independence. Gerontological researchers have identified that increased participation in social activities can lead to increased social connections among rural-dwelling older adults. Through educational programs, the Cooperative Extension (Extension) system connects older adults with opportunities to engage in community programs to reduce social isolation and support older adults living independently longer. Despite the widespread success of Extension programs, the implementation and collaborative efforts that contribute to the success of these programs are unrecorded in empirical literature. In this presentation, we address this gap by presenting on the Stay Strong Stay Healthy (SSSH) program. SSSH is as an example of the successful implementation of a multi-state Extension program that improves psychosocial and physical health outcomes for both rural and urban older adults. Using the SSSH program as a model, we outline the community-based research principles that inform the collaborations between Extension campus researchers, Extension agents, and stakeholders to reach rural-dwelling older adults. We will draw on a current example of adapting the SSSH program for less ambulatory older adults in rural areas guided by a community advisory committee. By leveraging the resources and expertise of Extension, researchers can enhance the real-world impact of their work and contribute to the reduction of social isolation and increased ability to live independent for rural-dwelling older adults.

